# Marked neurotropism and potential adaptation of H5N1 clade 2.3.4.4.b virus in naturally infected domestic cats

**DOI:** 10.1080/22221751.2024.2440498

**Published:** 2024-12-09

**Authors:** Shubhada K. Chothe, Surabhi Srinivas, Sougat Misra, Noel Chandan Nallipogu, Elizabeth Gilbride, Lindsey LaBella, Swastidipa Mukherjee, Christian H. Gauthier, Heidi L. Pecoraro, Brett T. Webb, James M. Pipas, Santhamani Ramasamy, Suresh V. Kuchipudi

**Affiliations:** a Department of Infectious Diseases and Microbiology, School of Public Health, University of Pittsburgh, Pittsburgh, PA, USA; b Center for Vaccine Research, University of Pittsburgh, Pittsburgh, PA, USA; c Department of Biological Sciences, University of Pittsburgh, Pittsburgh, PA, USA; d Veterinary Diagnostic Laboratory, North Dakota State University, Fargo, ND, USA

**Keywords:** Influenza A viruses, A(H5N1), clade 2.3.4.4b, neurotropism, influenza A virus evolution, avian influenza, cat

## Abstract

In April 2024, ten cats died in a rural South Dakota (SD) residence, showing respiratory and neurological symptoms. Necropsy and laboratory testing of two cats confirmed H5N1 clade 2.3.4.4b infection. The viral genome sequences are closely related to recent SD cattle H5N1 sequences. Cat H5N1 genomes had unique mutations, including T143A in haemagglutinin, known to affect infectivity and immune evasion, and two novel mutations in PA protein (F314L, L342Q) that may affect polymerase activity and virulence, suggesting potential virus adaptation. Dead cats showed systemic infection with lesions and viral antigens in multiple organs. Higher viral RNA and antigen in the brain indicated pronounced neurotropism. Lectin-histochemistry revealed widespread co-expression of sialic acid α-2,6 and α-2,3 receptors, suggesting cats could serve as mixing vessels for reassortment of avian and mammalian influenza viruses. No differences in clade 2.2 or 2.3.4.4b H5 pseudoviruses binding to cat lung/brain tissues indicated the neurotropism is unlikely mediated by receptor binding affinity.

## Introduction

Since the first discovery of the highly pathogenic avian influenza virus (HPAIV) of H5N1 subtype from a goose in Guangdong, China, in 1996, the virus has diversified into ten clades (Clades 0–9) and subclades [1]. In 2020, the H5N8 strain belonging to the 2.3.4.4b clade caused infections among domestic and wild birds across Europe, Africa, and Asia, leading to new reassortant strains like H5N1 clade 2.3.4.4b [2]. Since the first detection in the Netherlands in October 2020, H5N1 clade 2.3.4.4b virus outbreaks have been reported in multiple European countries, Africa, Asia, and America [3]. The geographic spread and the range of species affected by the clade 2.3.4.4b H5N1 viruses far exceed those of previous H5N1 clades. H5N1 clade 2.3.4.4b infections have been reported in over 90 species of wild and domestic birds and more than 21 mammalian species, including cattle, foxes, skunk, sea lions, mink, dolphins, raccoon dogs, cats, and seals [4–10], including several human infections [11]. Most mammalian infections likely occurred through ingestion or direct contact with infected birds, exacerbated by widespread infection and mortality of avian species [12]. The unprecedented risk of H5N1 clade 2.3.4.4b is highlighted by the massive deaths among sea lions between January and October 2023 across Peru, Chile, Argentina, Uruguay, and Brazil [6].

Animals infected with clade 2.3.4.4b H5N1 viruses commonly exhibited pneumonia and meningoencephalitis, with neurological signs predominating in several animal species. Notably, dolphins [8], skunks [13], minks [14], red foxes [15], and sea lions [6] showed significant neurological signs such as tremors, convulsions, and ataxia, with viral presence mainly in the brain. Though neurotropism and neurological signs were observed during the outbreaks of previous clades of H5N1viruses, the pronounced neurotropism of the current H5N1 clade 2.3.4.4b is highlighted by high viral loads in the brain and minimal or no viral presence in the lungs of several species [8,16], suggesting a significant shift in virus behaviour.

There have been 21 documented cases of H5N1 clade 2.3.4.4b infection in cats in the USA since March 2024 [17]. The susceptibility of cats to avian influenza H5N1 viruses became known in 2004. The first documented case of natural H5N1 infection in cats was in 2004 in Thailand, where fourteen cats died in a household [18]. At the same time, severe pneumonia in tigers and leopards fed on infected poultry carcasses in Thailand extended the susceptible host range of H5N1 viruses [19]. Experimental infection of cats with the H5N1 isolate from a fatal human case in Vietnam (clade 2.2 – A/Vietnam/1194/04) revealed that while the virus caused lower respiratory tract disease, it resulted in the death of only one out of three infected cats. Notably, there was no evidence of neurological deficits [20]. A subsequent report from Germany on the natural infection of domestic Shorthair cats with HPAIV H5N1 A/swan/Germany/R65/06 showed a higher viral load in the lungs compared to the brain, with 3.5 logs more virus in the lungs, associated with broncho-interstitial pneumonia [21]. Earlier H5N1 clades in cats led to either subclinical infections [22] or clinical disease characterized by pneumonia and encephalitis [23], and the currently circulating reassortant clade 2.3.4.4b H5N1 virus has also been heavily linked to respiratory and neurological signs in cats in France [24], Poland [25], South Korea [26], and the USA [4]. In this study, we report marked neurotropism of clade 2.3.4.4b H5N1 viruses in cats with an in-depth investigation of the histopathological lesions and viral detection in multiple organs.

A key determinant of the host susceptibility and tissue tropism of influenza viruses is the ability of the virus haemagglutinin (HA) to bind to host sialic acid (SA) receptors [27]. While human and other mammalian influenza viruses show preferential binding to SA receptors with α-2,6Gal linkage, avian influenza viruses, including H5N1, show preferential tropism to SA receptors with α-2,3Gal linkage [28]. The distribution of these receptors varies among species; for example, human upper respiratory tract epithelial cells mainly contain SA α-2,6Gal receptors [29], whereas SA α-2,3Gal is the predominant receptor in the trachea of duck and all species of Passeriformes [30, 31]. A shift in the binding affinity of the H5N1 virus's HA from SA α-2,3 to α-2,6-receptors is crucial for it to gain the ability of human-to-human transmission [32]. Several amino acid changes (N182 K, Q222L/G224S, or the combination N182 K/Q222L/G224S) in the H5 protein have been identified that enhance binding to SA α-2,6-receptors [32]. A recent study reported that clade 2.3.4.4b viruses recovered from mesocarnivores in Canada retained amino acid residues in their HA protein that may enable binding to SA α-2,6-receptors. Notably, the substitutions S137A and T160A were shown *in vitro* to moderately enhance influenza A virus (IAV) binding to SA α-2,6 receptors [33]. The neurotropism of clade 2.3.4.4b H5N1 viruses in cats could be due to differences in receptor distribution patterns. We conducted an in-depth evaluation of SA receptor distribution across multiple organs in cats and found widespread co-expression of avian and human influenza receptors across multiple cat tissues, with no notable differences between the lung and the brain. These observations supplement a previously published report that showed tissue distribution of human and avian-type sialic acid influenza virus receptors in domestic cats [34]. Further, we found widespread binding of pseudoviruses expressing H1 and H5 from clade 2.2 and 2.3.4.4b to cat tissues. Further, no notable differences in the binding patterns of clade 2.2 and clade 2.3.4.4b to cat lung and brain tissues suggest that neurotropism of clade 2.3.4.4b viruses is unlikely mediated by receptor binding affinity.

## Materials and methods

### Animal tissues

Two dead cats, aged six months and one and a half years, were necropsied at the North Dakota Veterinary Diagnostic Laboratory (NDSU VDL) in the month of April 2024. Clinical signs in these cats include reduced appetite, lethargy, and neurological impairments, which lead to mortality. A comprehensive set of tissues including lung, kidney, heart, stomach, ileum, jejunum, duodenum, colon, pancreas, adrenal gland, cerebrum, cerebellum, hippocampus, brainstem, skeletal muscle, thyroid/parathyroid, lymph node, liver, and spleen were fixed in 10% neutral buffered formalin. The fixed tissues underwent routine histological processing and sectioning for haematoxylin and eosin (H&E) staining and immunohistochemical analysis. Lung and brain tissues were collected for nucleic acid extraction and PCR. Formalin-fixed, paraffin-embedded healthy cat tissue sections were acquired from Zyagen in San Diego, California, USA. These included lung (FP-601), cerebral cortex (FP-210), stomach (FP-302), ileum (FP-309), jejunum (FP-308), and duodenum (FP-307).

### Quantitative RT–PCR and next-generation sequencing

The RNA extraction and IAV matrix/H5 PCR were conducted at the NDSU VDL following the NVSL-approved protocols. The RNA samples were subjected to next-generation sequencing at USDA Animal Plant Health Inspection Service-National Veterinary Services Laboratories. The paired-end whole genome sequencing was performed using the NextSeq 2000 Illumina instrument. The sequence reads were submitted to SRA, NCBI database (SRR29040272, SRR29040273), and the corresponding complete gene segments assembled using Geneious Prime version 2024.0.4 were submitted to GISAID (EPI_ISL_19196362 and EPI_ISL_19196363).

### Phylogenetic analysis

H5N1 HA sequences derived from the two cats in this study were analysed with a total of 1443 HA sequences belonging to the H5N1 clade 2.3.4.4b that were downloaded from GISAID on June 7, 2024. These sequences originated from humans (global), chicken (North America), duck, goose, and all mammals from North and South America, including the recently reported sequences originating from infected dairy cattle. Duplicate sequences with identical nucleotide composition were removed from the analyses, yielding a total of 745 sequences available for the construction of a phylogenetic tree. Sequences were aligned using the following parameters: gap open cost = 10 and gap extension cost = 1.0 with accurate alignment mode (CLC Genomics Workbench, version 24). The coding sequence of HA from all the sequences was extracted from the alignment and subjected to nucleotide substitution testing employing the following models: Jukes-Cantor [35], Felsenstein81 [36], Kimura80 [37], Hasegawa-Kishino-Yano (HKY) [38], and General Time Reversible (GTR) (also known as the REV model) [39]. The outlined nucleotide substitution models were tested using the Hierarchical likelihood ratio test (hLRT), Bayesian information criterion (BIC), Minimum theoretical information criterion (AIC, Akaike information criterion), and minimum corrected theoretical information criterion (AICc, AIC with a correction for the sample size) to identify the best model for phylogenetic tree construction. The phylogenetic tree for the HA gene was constructed using the neighbor-joining method employing the GTR nucleotide substitution model, which yielded the best model. Rate variation was included in the maximum likelihood phylogeny analysis with the number of substitution rate categories set to 4, and the gamma distribution parameter was set to 1.0. Bootstrap analysis was performed with 100 replicates that afforded computationally feasible phylogenetic tree construction.

### Structure prediction and visualization

The monomeric and multimeric structures of the feline influenza virus proteins were predicted with AlphaFold version 2.3.2, using databases downloaded on March 24, 2024. For both monomers and multimers, AlphaFold was run using the full genetic databases (–db_preset=full_dbs), with relaxation enabled on the best-scoring model (–models_to_relax=best) and templating disabled (–max_template_date = 1900-01-01). AlphaFold-predicted structures were visualized in PyMOL 2.5.4. The “super” command was used to superposition comparable structures from PDB.

### Lectin histochemistry

Lectin histochemistry was carried out on paraffin-embedded tissue sections as previously described [31]. Briefly, sections of 5 µm thickness were first deparaffinized in xylene and rehydrated through a series of ethanol treatments. The rehydrated sections underwent a 5-minute pre-soak in Tris-buffered saline (TBS), followed by antigen retrieval using eBioscience™ IHC Antigen Retrieval Solution – Low pH (10X) (ThermoFisher Scientific, catalog# 00-4955-58), according to the manufacturer's instructions. Sections were then blocked using goat serum (Thermo Fisher Scientific, catalog# PCN5000) diluted 1:40 with TBS and further blocked using a Streptavidin/Biotin Blocking Kit (Vector Laboratories, SP2002), following the manufacturer's guidelines. The sections were incubated with *Sambucus Nigra* Lectin (SNA), Fluorescein-labeled (Vector Laboratories, FL13012), *Maackia Amurensis* Lectin II (MAA II), Unconjugated (Vector Laboratories, L-1260-2), and *Maackia Amurensis* Lectin I (MAA I), Fluorescein (Vector Laboratories, FL13112). SNA binds specifically to sialic acid linked to galactose via an alpha-2,6 linkage (SA α-2,6-Gal), while MAL II and MAL I bind to sialic acid via an alpha-2,3 linkage (SA α-2,3-Gal), while MAL I has a preference for distinct glycan structures compared to MAL II. Each lectin was used at a concentration of 10 μg/ml and incubated overnight at 4°C. After three TBS washes, sections were treated with Streptavidin, Alexa Fluor 594 Conjugate (Thermo Fisher Scientific, catalog# S32356) for 2 h at 4°C to stain the lectins. Following three washes with TBS, sections were mounted using ProLong™ Gold Antifade Mountant with DAPI (Thermo Fisher Scientific, catalog# P36931). After curing for 24 h at room temperature, the sections were imaged with an Echo Revolve Fluorescent Microscope. Negative control staining, performed by omitting the lectins, showed minimal background (Supplementary Figure 5).

### Influenza pseudovirus production

The 3rd generation lentiviral plasmids obtained from BEI resources (catalog # NR-53816) were used to produce H5 and H1 pseudotyped viruses. The influenza A H5N1 HA sequences of clade 2.2 and clade 2.3.4.4b were retrieved from NCBI, and expression constructs were designed using SnapGene software. The constructs were synthesized by GenScript, USA, and cloned into a pHCMV vector under the control of a CMV promoter. H1 expression plasmid. pHCMV_FLUAV_HA was a gift from Feng Zhang (Addgene plasmid # 207273; http://n2t.net/addgene:207273; RRID: Addgene_207273) [40]. Lentiviral helper plasmids, reporter plasmids, and plasmids encoding H5 or H1 were co-transfected into HEK 293 T cells using Fugene6 transfection reagent (Promega, USA, catalog # E2691). Pseudoviruses were collected 48 h post-transfection, and titres were determined by infecting the HEK-293 T cells and measuring the luciferase expression using BrightGlo reagent (Promega, USA, catalog # E2650). The relative luminescence units (RLU) were measured using BioTek Cytation 7 Cell Imaging Multimode Reader. The pseudoviruses with infectivity titre between 10^4^ and 10^5^ RLU were used for the virus binding assay.

### Virus binding assay

A virus binding assay was conducted using Influenza A pseudoviruses (H5N1 HA, clade 2.2; H5N1 HA, clade 2.3.4.4b; and H1). The deparaffinized and antigen-retrieved tissue sections were incubated with 250μl of Influenza A pseudoviruses at 37°C for 2 h. The RLU of H5 clade 2.3.4.4b, H5 clade 2.2, and H1 pseudoviruses used in the virus binding assay were 109591, 88,124, and 39,879, respectively. Further, the sections were blocked with inactivated goat serum (Thermo Fisher Scientific, catalog # PCN5000) at a 1:40 dilution overnight. The sections treated with H5N1 were immunostained using a primary mouse monoclonal anti-Influenza Virus H5 Haemagglutinin (HA) Protein (VN04-8), A/Vietnam/1203/2004 (H5N1) from BEI resources (NR-2733), while those treated with H1 were stained with anti-Influenza A H1N1 haemagglutinin antibody [C102 (IV.C102)] (Abcam, catalog # ab128412), both at a 1:100 dilution for 90 min. Post-primary antibody incubation, the sections were washed and incubated with a secondary goat anti-mouse IgG H&L (Alexa Fluor® 647) antibody (Abcam, catalog # ab150115) at a 1:800 dilution for 35 min. After three TBS washes, the sections were mounted in ProLong Gold antifade mountant with DAPI. Following a 24-hour curing period at room temperature, the sections were imaged using an Echo Revolve Fluorescent Microscope. Negative control staining, performed by omitting the virus and primary antibody, showed minimal background (Supplementary Figure 5). Control experiments were conducted by substituting the influenza pseudovirus with a SARS-CoV-2 pseudovirus, demonstrating no cross-reactivity in the staining (data not shown).

### Immunohistochemistry for the detection of influenza A virus nucleoprotein

Tissue sections were processed as mentioned in the lectin histochemistry methodology. Following antigen retrieval, the sections were blocked with goat serum (Thermo Fisher Scientific, catalog # PCN5000) diluted 1:40 in TBS. Next, the sections were incubated with primary anti-Influenza A virus nucleoprotein antibody, Isotype: IgG2a (1:100) (Abcam, catalog # ab20343) for 90 min, followed by secondary goat anti-mouse IgG H&L (Alexa Fluor® 647) antibody (Abcam, catalog # ab150115) at a 1:800 dilution for 35 min. After three TBS washes, the sections were mounted in ProLong Gold antifade mountant with DAPI. After a 24-hour curing period at room temperature, the sections were imaged using an Echo Revolve Fluorescent Microscope. Negative control staining, performed by omitting the primary antibody, showed minimal background (Supplementary Figure 5). Isotype control staining was performed using Mouse IgG2a kappa Isotype Control (eBM2a), eBioscience (Thermo Fisher Catalog # 14-4724-82), which showed no cross-reaction and minimal background staining (data not shown).

### Fluorescence intensity quantification

The LHC and IHC images were subjected to quantification of the fluorescence using the software ImageJ (v 1.53). For each tissue section, fluorescence intensity was measured at five distinct sites of identical size within the image, and the mean value of these measurements was calculated to represent the mean fluorescence intensity. For intestinal tissues, the region of interest was focused on the mucosal lining, as this area is likely to come into contact with the virus upon ingestion. This approach ensured consistency in quantification across all tissue types analysed. The mean fluorescence intensity values were statistically analysed using one-way ANOVA, followed by post hoc Tukey's test to determine significant differences between groups.

## Results

### Outbreak of H5N1 clade 2.3.4.4b virus in outdoor cats in rural South Dakota

In a rural residential area in South Dakota (SD), ten outdoor-housed cats aged 6 months to 4 years were found deceased. Most of these cats were domesticated and regarded as family pets. Clinical signs included anorexia, lethargy, and potential neurologic deficits. Two deceased cats, aged six and eighteen months, were submitted to the North Dakota State University Veterinary Diagnostic Laboratory (NDSU VDL) for postmortem examinations. Along with positive *Streptococcus canis* respiratory cultures, Polymerase Chain Reaction (PCR) results revealed the presence of influenza A virus (IAV) in the brain and lungs. The Cycle threshold (Ct) values for the IAV matrix gene in the brain were 20.1 and 18.1, and in the lungs were 35.93 and 31.93 for cats 1 and 2, respectively, indicating much higher viral load in the brains of both cats. Samples were submitted to the National Veterinary Services Laboratory (NVSL), Ames, Iowa, and further confirmed to be H5N1 clade 2.3.4.4b by PCR and whole genome sequencing**.**

### Unique mutations in H5N1 clade 2.3.4.4b sequences from fatal cat cases in SD

**Figure 1.**
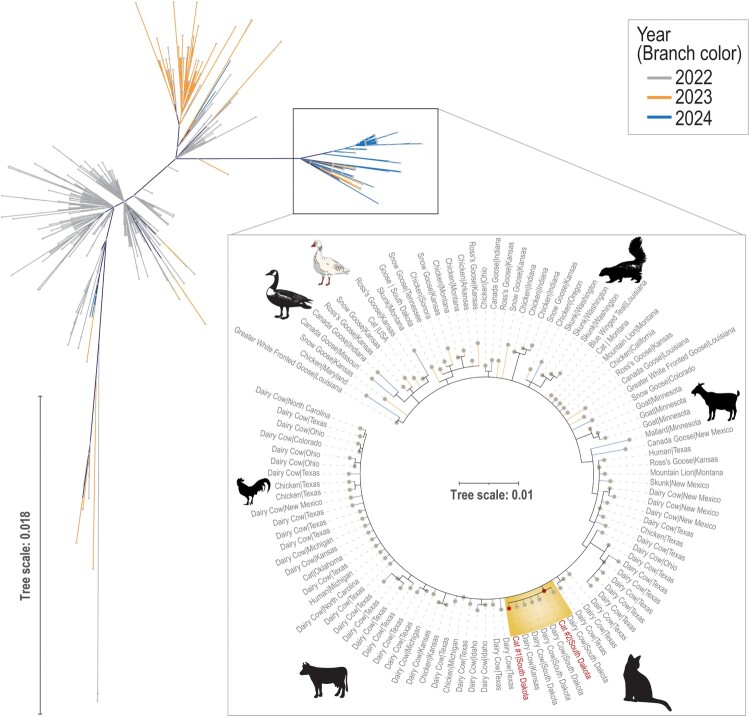
Maximum likelihood-based haemagglutinin (HA) phylogenetic tree originating from 1443 sequences of HPAIV H5N1, clade 2.3.4.4b. The highlighted sub-branch of the unrooted tree contains the HPAIV H5N1 clade 2.3.4.4b sequences from two cats from South Dakota.

A phylogenetic tree of the HA gene from the two cat H5N1 clade 2.3.4.4b sequences was constructed alongside 1443 H5N1 clade 2.3.4.4b HA sequences from humans worldwide, chicken (North America), duck, goose, and all mammals from North and South America. All statistical tests indicated that the inclusion of rate and topology variation in the GTR model yielded the best-fit nucleotide substitution model (Supplementary Tables 2–4). The resulting maximum likelihood phylogeny tree showed that the HA gene from the South Dakota HPAIV H5N1 clade 2.3.4.4b cat sequences were closest to the H5N1 clade 2.3.4.4b sequences from dairy cattle samples originated from South Dakota and Kansas (Figure 1, annotated highlighted area).

Further, eight major IAV protein sequences of the cat H5N1 were analysed alongside 178 dairy cattle sequences and 13 cat sequences obtained from the GISAID database[41]. Sequence alignment was performed using CLC Genomics Workbench, v24. The consensus sequence from the alignment was used as a benchmark to detect the occurrence of mutations in the eight proteins. Two unique mutations were found in the HA (T143A) and neuraminidase (NA) (N71S) proteins. One of the cats H5N1 sequences (GISAID ID # EPI_ISL_19196362) had a mutation in the polymerase acidic (PA) protein (F314L), whereas L342Q mutation was detected in the sequence originating from the other cat (GISAID # EPI_ISL_19196363). There was no evidence of mutations in other proteins.

### Structural and functional implications of HA, NA, and PA mutations in the cat H5N1 sequences

**Figure 2.**
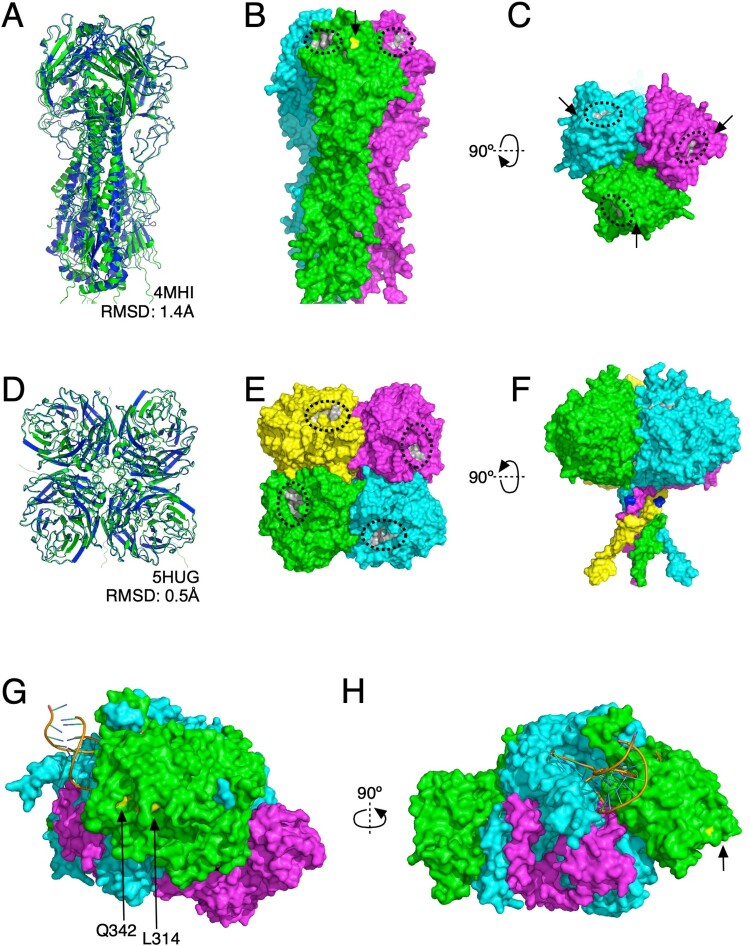
Exploration of mutational consequences on protein structure. (A) Side-view cartoon representation of the AlphaFold-predicted structure for HA trimer (green) superposed with a solved structure (PDB code: 4MHI; blue). (B) Surface representation of the predicted HA trimer structure, with subunits coloured in green, cyan, and magenta. The sialic acid binding pocket is circled and shaded grey near the top of the receptor-binding domain, and the nearby T143A mutation is in yellow with an arrow pointing to it. (C) The same but rotated 90 degrees to view from above the receptor-binding domain; all three sialic acid binding pockets and T143A positions are indicated. (D) Top-down view cartoon representation of the AlphaFold-predicted structure for NA tetramer (green) superposed with a solved structure (PDB code: 5HUG; blue). (E) The surface representation of the predicted NA tetramer structure, with subunits coloured green, cyan, magenta, and yellow. The sialic acid binding pocket is circled and shaded grey. (F) The same but rotated 90 degrees to view from the side; the location of the N71S mutation is indicated in blue. (G) Side-view surface representation of a viral polymerase complex previously solved by X-ray crystallography (8H69); the PA, PB1, and PB2 subunits are shaded green, cyan, and magenta, respectively. The locations of the mutations we observed in PA are shaded yellow. (H) The same but rotated 90 degrees to highlight the distance of observed mutations from the catalytic core of the polymerase complex.

HA Mutation: The top-ranked AlphaFold model of the trimer had very good agreement with PDB structure 4MHI (RMSD = 1.4 Å). Most of the variance manifested as differentially pitched or bent alpha helices in the stalk of HA; the receptor-binding domain was predicted to adopt an essentially identical conformation to the solved structure (Figure 2(A)). Amino acid 143 was positioned very close to the SA binding site (Figure 2(B,C)).

NA Mutation: The top-ranked AlphaFold model of the tetramer showed near-perfect agreement with PDB structure 5HUG (RMSD = 0.5 Å) (Figure 2(D)). The N71S mutation occurred in the stalk between the catalytic head and the transmembrane helices (Figure 2(E,F)), far from the sialic acid binding sites and in a region not modelled in the X-ray diffraction data.

PA Subunit Mutations: The two recovered feline influenza viruses had a single substitution in the PA subunit of the viral polymerase. One had an F314L substitution, and the other had an L342Q substitution, both located on the outside of the polymerase complex, far from the catalytic core (Figure 2(G–H)).

### Severe neuropathology with extensive viral nucleoprotein in the brains of cats infected with H5N1 clade 2.3.4.4b

**Figure 3.**
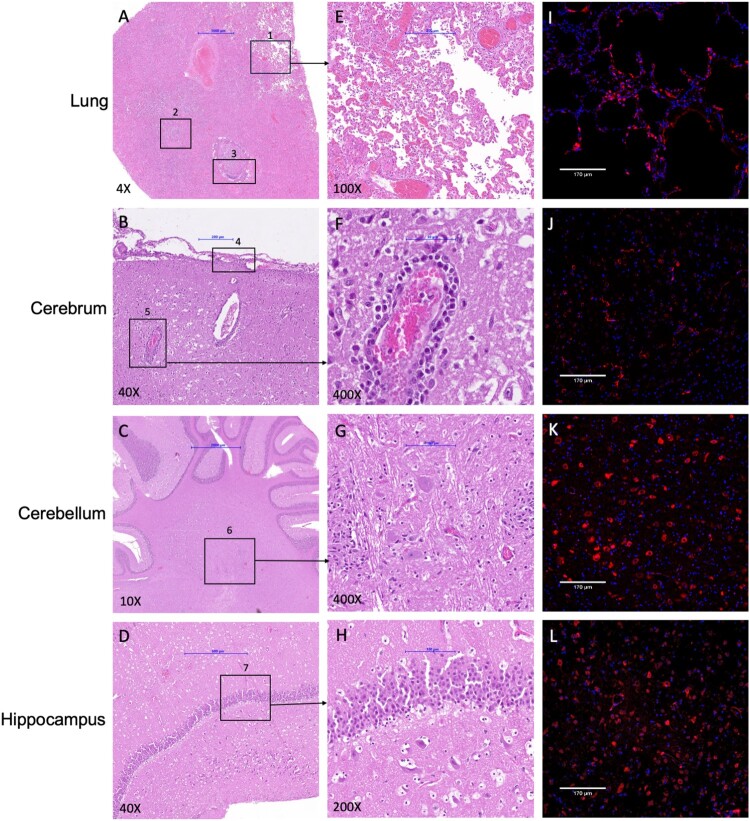
Histopathological and immunohistochemical analysis of HPAIV H5N1 infected cat tissue sections. H&E-stained tissue sections of HPAIV H5N1 infected cat showed various histologic lesions. (A) Lung exhibited (1) interstitial pneumonia, (2) bronchiolitis, (3) bronchitis. (B) Cerebrum had (4) meningitis and (5) encephalitis whereas (D) hippocampus showed no lesions. The immunohistochemical (IHC) analysis (I-L) revealed the presence of IAV nucleoprotein in each of these organs. Brain tissues showed a higher nucleoprotein staining level than the lung tissue, with the cerebellum and hippocampus exhibiting extensive presence of nucleoprotein. Tissues were primarily stained using an anti-influenza A virus nucleoprotein antibody, followed by a secondary goat anti-mouse IgG H&L (Alexa Fluor® 647) antibody (red) and DAPI nuclear stain (blue). The H&E-stained images were acquired by Epredia Pannoramic MIDI II using Z stacking with extended focus, and the IHC images were acquired by ECHO revolve microscope.

We performed a detailed histopathological investigation of the tissues, including lung, kidney, heart, stomach, ileum, jejunum, duodenum, colon, pancreas, adrenal gland, cerebrum, cerebellum, hippocampus, brainstem, skeletal muscle, thyroid/parathyroid, lymph node, liver, and spleen. Supplementary Table 1 details the histopathological lesions found in various organs of cats. Briefly, microscopic evaluation of lung sections revealed mixed mononuclear and neutrophilic inflammatory cells in alveoli with fibrin deposition and oedema, alveolar epithelial necrosis, alveolar hyaline membranes, bronchiolitis, bronchiolar epithelial hyperplasia, perivasculitis, and perivascular oedema. Bronchitis was observed in one of the cats. Within the brain was mononuclear perivascular cuffing in the cerebellum, brain stem, and cerebrum with random foci of necrosis and suppurative inflammation, gliosis, neuronal necrosis, oedema, and perivasculitis. Mild mononuclear perivascular infiltrates in the submucosa and tunica muscularis of the small and large intestines were observed in both cats. Aggregates of mixed mononuclear cells and neutrophils and necrosis in the ventricular sub-endomyocardium, as well as random foci of hepatitis, were found in one of the cats. The pancreas (exocrine and ductular regions) and thyroid gland were multifocally expanded by mild to moderate mononuclear infiltrates. The spleen showed marginal hyperaemia and follicular lymphoid depletion. Increased numbers of tingible-body macrophages were seen in the cortex and medullary sinus of the lymph node. Figure 3(A–H) shows the H&E-stained images of the histologic lesions observed in the cats.

We further conducted immunohistochemistry on the tissues, targeting the IAV nucleoprotein, which revealed intense staining in a higher proportion of cells in various parts of the brain and intestine. Noticeably, the cerebellum and the hippocampus showed an abundance of nucleoprotein compared to the lungs (Figure 3(I–L)), indicating a higher viral load in brain tissues, as confirmed by the PCR assay. Virus nucleoprotein was also found in other organs of the cat, particularly in the jejunum and colon (Supplementary Figure 1).

### Abundant co-expression of SA α-2,3-Gal and SA α-2,6-Gal receptors in cat brain and lung

**Figure 4.**
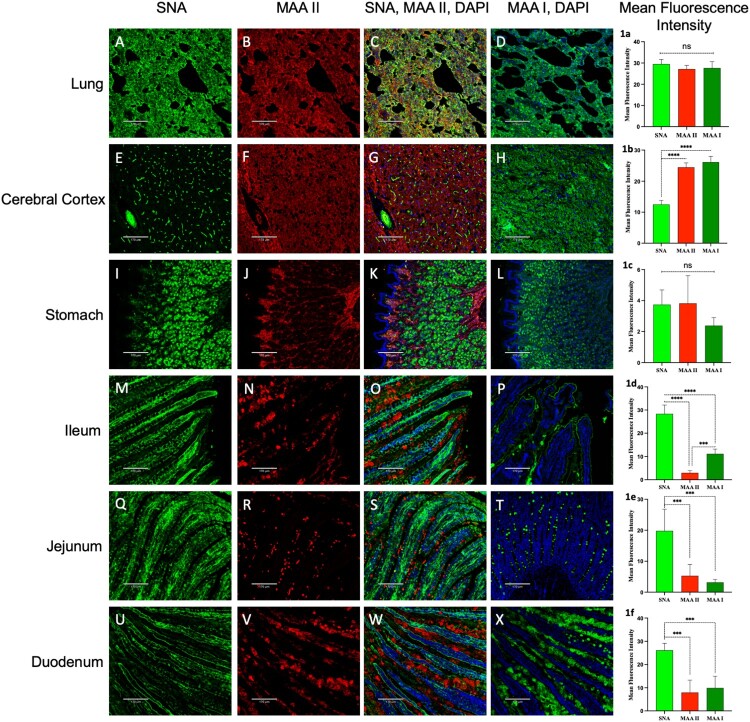
Co-expression of SA α-2,3-Gal and SA α-2,6-Gal receptors in cat tissues. The composite fluorescent images reveal co-expression of SA α-2,3-Gal (red) and SA α-2,6-Gal (green) influenza receptors in the lung, cerebral cortex, stomach, ileum, jejunum, and duodenum of the cat. The lung and cerebral cortex exhibit co-expression of these receptors, whereas the gastrointestinal tissues display a higher expression of SA α-2,6-Gal (green) receptors, particularly on the luminal mucosal lining of the ileum, jejunum, and duodenum. Tissue sections were stained with FITC-labeled SNA (SA α-2,6-Gal) lectin, FITC-labeled MAA I (SA α-2,3-Gal), biotinylated MAA II (SA α-2,3-Gal), and DAPI nuclear stain (blue). Panels 1a–1f present the quantified mean fluorescence intensity for each corresponding tissue. Bars represent the mean ± standard deviation (SD) for each group (***p < 0.001, ****p < 0.0001). Mock-treated images are available in the supplementary data. Scale bar = 170 µm.

Significant co-expression of avian (SA α-2,3-Gal) and human (SA α-2,6-Gal) type influenza -receptors was observed in both healthy (Figure 4) and infected cat tissues (Supplementary Figures 2–4). SNA staining indicated a widespread presence of α-2,6-linked sialic acids, while MAL I and MAL II lectins provided detailed insights into sialylation patterns: MAL I detected simpler sialylated structures with α-2,3-linked sialic acids [SA α-2,3-Gal-β (1–4) N-acetylglucosamine] and MAL II highlighted complex sialylated structures with the same α-2,3 linkage [SA α-2,3-Gal-β (1–3) N-acetylgalactosamine (GalNAc)]. The abundant co-expression of SA α-2,3-Gal and SA α-2,6-Gal was particularly notable in healthy cats’ cerebral cortex (Figure 4(E-H, and 1b)). Lung tissue from healthy cats also exhibited a high abundance of both avian and human-type influenza receptors, especially in the alveolar epithelium, alveolar duct, and visceral pleura of the lung (Figure 4(A–D)) with no significant quantitative difference within the receptors (Figure 4, 1a). In the brain tissues of infected cats, including the cerebellum, cerebrum, brainstem, and hippocampus (Suppl. Fig. 2), co-expression of SA α-2,3-Gal and SA α-2,6-Gal receptors was also observed.

### Predominant expression of SA α-2,6-Gal receptors in cat intestines

The stomach, ileum, jejunum, and duodenum of healthy cat tissue showed co-expression of SA α-2,3-Gal and SA α-2,6-Gal receptors (Figure 4, I-X and 1c-1f). Notably, the ileum, jejunum, and duodenum exhibited a predominance of SA α-2,6-Gal receptors on the mucosal lining of the villi (Figure 4, 1d-1f). These receptors were also present in the goblet cells, lamina propria, muscularis, and intestine serosa. In contrast, the avian-type receptors (SA α-2,3-Gal) were concentrated along the basal region of the villi, the underlying lamina propria, crypts, submucosal regions, and muscularis mucosae.

### Comparative binding of pseudoviruses with clades 2.2 H5 and clade 2.3.4.4b H5, and H1 to cat brain and lung tissues

**Figure 5.**
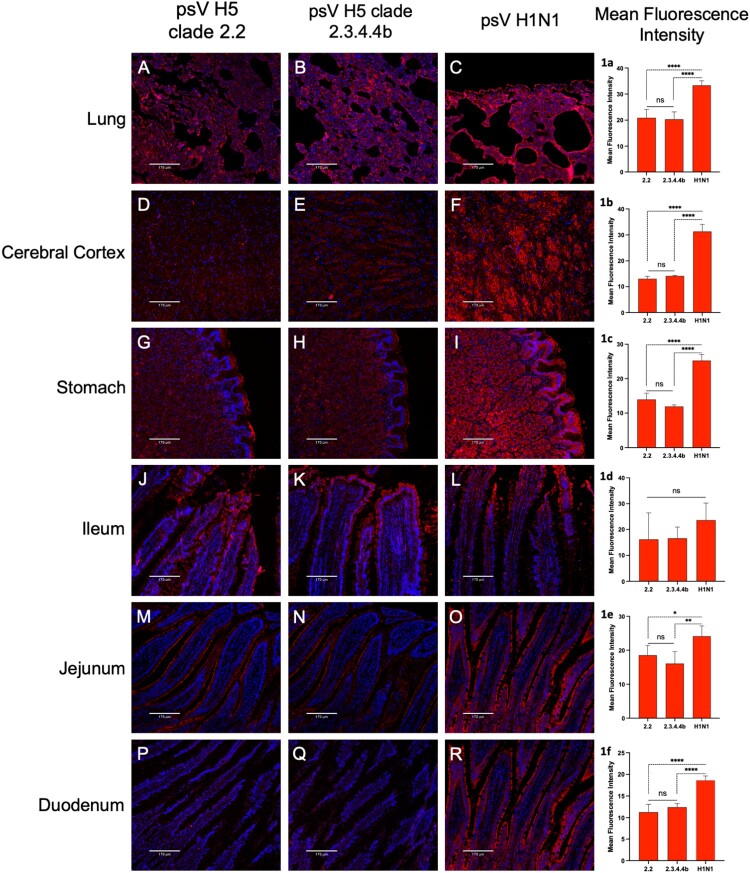
Widespread binding of pseudoviruses carrying H5 of clade 2.2 or clade 2.3.4.4b and human influenza H1 to cat tissues. The fluorescent images illustrate the extensive binding of H5N1 clade 2.2, 2.3.4.4b and human H1 pseudoviruses to cat tissues, including the (A,B,C) lung; (D,E,F) cerebral cortex; (G,H,I) stomach; (J,K,L) ileum; (M,N,O) jejunum; and (P,Q,R) duodenum. This binding pattern correlates with the relative abundance of SA α-2,3-Gal and SA α-2,6-Gal receptors in these tissues. The mucosal endothelial cells lining the alveoli and the luminal mucosal cells of the intestine exhibit a strong preference for virus binding. The tissue sections were primarily stained with mouse monoclonal anti-influenza virus H5 haemagglutinin (HA) protein (VN04-8), A/Vietnam/1203/2004 (H5N1) or anti-influenza A H1N1 haemagglutinin antibody followed by secondary goat anti-mouse IgG H&L (Alexa Fluor® 647) antibody (red) and DAPI nuclear stain (blue). Panel 1A-1F shows the quantified mean fluorescence intensity for each virus binding. Bars represent the mean ± standard deviation (SD) for each group (*p < 0.05, **p < 0.01, ****p < 0.0001). Mock-treated images are provided in the supplementary data. Scale bar = 170 µm.

Virus–receptor binding assays were performed on healthy cat lungs, cerebral cortex, and gastrointestinal tissues (stomach, ileum, jejunum, and duodenum) using pseudoviruses expressing H1 or H5 from H5N1 clade 2.2 or H5N1 clade 2.3.4.4b generated in our laboratory. The cat SA α-2,3-Gal and SA α-2,6-Gal receptors were found to be compatible with binding to the pseudoviruses, and the virus binding pattern correlated with the relative abundance of the receptors, with greater receptor expression, leading to greater virus binding. The H1 pseudovirus exhibited abundant binding to various tissues, reflecting the high abundance of SA α-2,6-Gal receptors in cats. Both the H5 pseudoviruses (clade 2.2 and clade 2.3.4.4b) exhibited binding to multiple tissues; binding was less extensive than the H1 pseudovirus (Figure 5). No significant difference in binding was observed between pseudoviruses carrying clade 2.2 H5 and clade 2.3.4.4b H5 (Figure 5, 1a-1f). The H1 and H5 pseudovirus bound extensively to the mucosal lining of the intestines, indicating the potential for enteric pathogenesis upon oral infection.

## Discussion

Increasing evidence suggests recent shifts in the patterns of mammalian infections with the HPAI H5N1 viruses worldwide, indicating ongoing adaptation to infect mammalian hosts [42] In addition, the host range of the HPAIV H5N1 virus has been expanding, with clade 2.3.4.4b spillovers now detected in various mammalian species. These include both domestic and wild carnivores, such as domestic cats [43], red foxes [44], multiple bear species [45], and seals [10] among others. This growing list of susceptible mammalian hosts highlights the virus's ability to cross species barriers, raising concerns about its potential impact on wildlife and domestic animal populations.

In this study, we report a natural H5N1 clade 2.3.4.4b virus infection resulting in the deaths of ten cats in rural South Dakota. The exact source of infection remains unclear; however, phylogenetic analysis of H5N1 sequences from two of the cats reveals a close genetic relationship to clade 2.3.4.4b strains previously detected in local cattle, suggesting a possible link. Additionally, the presence of bird feathers near the deceased cats indicates the likelihood that infection may have occurred through the consumption of virus-infected birds. However, because the disease typically requires several days to manifest post-ingestion, the exact timing of exposure is unclear. This evidence points toward a plausible cattle-to-bird-to-cat transmission pathway, supported by recent studies that identified H5N1 sequences across multiple species on affected farms, including dairy cows, wild birds, domestic cats, and raccoons [46].

Our study provides a significant new insight into the neurotropism of the H5N1 clade 2.3.4.4b virus in naturally infected domestic cats. There is a notable shift in the neurotropism of HPAI H5N1 viruses, particularly with the emergence of clade 2.3.4.4b in cats and wild carnivores like foxes. For example, in cats, experimental infection with the H5N1 Vietnam isolate (clade 2.2 – A/Vietnam/1194/04) showed primarily respiratory disease, with only one of three infected cats succumbing and no neurological symptoms [20]. Similarly, a natural H5N1 infection in Germany (A/swan/Germany/R65/06) in domestic cats displayed higher viral loads in the lungs than in the brain, with infection linked mainly to broncho-interstitial pneumonia [21]. Furthermore, studies on red foxes fed bird carcasses infected with clade 2.2 H5N1 also demonstrated limited clinical impact, with the foxes excreting the virus without developing severe disease. In contrast, recent H5N1 clade 2.3.4.4b infection in two cats from Texas [47] resulted in neurological signs and 50% mortality, likely due to ingestion of unpasteurized milk from infected cattle. Further, recent reports from Europe and the United States involving infection of red foxes with H5N1 clade 2.3.4.4b have shown a marked shift toward neurotropism [44]. These cases have documented viral adaptations that facilitate central nervous system involvement, with some infections exhibiting viral mutations indicative of enhanced neurotropism [46].

We identified several key mutations in the H5N1 sequence from infected cats that may suggest adaptation to cats. We observed a threonine-to-alanine mutation at residue 143 in HA (T143A). In A/Netherlands/219/2003 HA, this threonine (T143) forms a glycosylation motif (N-X-T/S) involving asparagine 141 (N141) around the receptor binding site (RBS), which is known to increase virus infectivity and resistance to neutralizing antibodies [48]. While the potential implications of the T143A mutation in clade 2.3.4.4.b are unclear, it could represent an adaptation mutation in cats that warrants further investigation. Notably, residue 143 in HA has been identified as a major mutation site in the RBS of H5 that contributes to viral escape from neutralizing antibodies among the different subclades, including 2.3.4.4b [49].

The N71S mutation in NA has not been previously reported in H5N1. While this mutation may not likely alter substrate specificity directly, it could potentially reduce efficiency on some substrates because phosphorylation or glycosylation of the serine residue might make the stalk of NA more rigid [50,51]. The PA mutations (F314L and L342Q) are novel, and their functional implications are unknown. However, it is important to note that mutations in residues adjacent to these positions (343 and 347) in avian H5N1 influenza viruses have been shown to affect polymerase activity and virulence in mice [52]. Therefore, further investigation of these two PA mutations is critical to better understand their impact on the virus's polymerase activity and mammalian pathogenicity.

Consistent with our findings, a 2023 study reported H5N1 clade 2.3.4.4b infection in cats in Poland with multi-organ lesions and higher viral load in the brain compared to the respiratory tract [53]. However, all the viruses from Poland had mutation PB2-E627 K, which is an important molecular marker of virus adaptation to mammals [53–55]. However, this mutation was absent in the H5N1 sequences from the cats in our study. The lack of PB2-E627 K in our cases suggests that alternative mechanisms may be driving the virus’s neurotropic behaviour, indicating a potentially unique adaptation pathway in these cats that warrants further investigation.

The co-expression of avian and mammalian SA receptors in cats identified in this study, combined with their potential exposure to various influenza viruses, poses a significant risk for genetic reassortment of different influenza strains, leading to the emergence of novel viral variants. A previous report examined the tissue distribution of human and avian-type sialic acid influenza virus receptors in cats, showing their expression in various organs [56]. Contrary to our findings, the earlier report indicated absence of alpha-2,3 sialic acid-linked receptors in the gastrointestinal tract and the absence of both alpha-2, 3 and alpha-2, 6 sialic acid-linked receptors in organs of the central nervous system, such as cerebrum and cerebellum. Further, unlike our study, the report did not investigate the binding patterns of MAA I (highly specific to SA α2,3 binding) binding receptors [31]. In the current study, we observed co-expression of SA α-2,3-Gal and SA α-2,6-Gal receptors in the lung, cerebral cortex, and gastrointestinal tissues. The luminal mucosal lining of the ileum, jejunum, and duodenum expressed high SA α-2,6-Gal (green) receptors, which is significant as this surface may come into contact with ingested viral particles. MAA I-specific SA receptors were also detected in the lungs, cerebral cortex, and lamina propria of gastrointestinal tissues. The limited use of lectins, variations in staining methodologies – such as immunochemical (previous study) vs. fluorescent staining (current study) – and differences in the number of animals investigated may have contributed to the discrepancies observed. Furthermore, other factors like sample processing conditions, individual variability among subjects, and differences in tissue collection and handling protocols could also play a role in the variations between our findings and those of previous studies.

In many rural households, as was the case with the infected cats in rural South Dakota reported in this study, cats are often housed outdoors, used for pest control, and considered family pets. This unique role exposes them to diverse environments and interactions, including terrestrial, aquatic, wild birds, and other livestock animals and humans. This exposure puts cats at a higher risk of encountering a broad spectrum of avian and mammalian influenza viruses. Notably, a recent study found that stray cats in the Netherlands were frequently exposed to HPAI H5, with a seropositivity rate of 11.8% among clinically healthy individuals [57]. The presence of asymptomatic infections in cats with H5N1 is a significant threat as these cats could serve as silent carriers, transmitting the virus to humans without showing any clinical signs of illness.

The continued exposure, viral circulation, and adaptation of the H5N1 virus in cats raise significant concerns for transmission and public health. Cats, common companion animals that frequently interact with humans and other species, could serve as a bridge for cross-species transmission of H5N1 viruses. Infected cats develop systemic infections and shed the virus through both respiratory and digestive tracts [58], potentially creating multiple routes of exposure to humans. Furthermore, the ability of the virus to persist and adapt in mammalian hosts heightens the risk of evolving into strains with increased transmissibility, posing an emerging zoonotic threat with profound public health implications. As H5N1 viruses continue to infect a wide range of avian and mammalian hosts, including an increasing number of human cases, there is an urgent need for coordinated One Health surveillance to monitor the spread of H5N1 among domestic and wild birds, animals, and humans.

## Supplementary Material

cat_H5N1_supplementary_data.pdf
