# Supplementary material for: Marked neurotropism and potential adaptation of H5N1 clade 2.3.4.4.b virus in naturally infected domestic cats
Source: Emerg Microbes Infect. 2024 Dec 9;14(1):2440498. doi: 10.1080/22221751.2024.2440498 (PMC11654043; doi:10.1080/22221751.2024.2440498)
Supplement: cat_H5N1_supplementary_data.pdf [file TEMI_A_2440498_SM1989.pdf]

Marked Neurotropism and Potential Adaptation of H5N1 Clade 2.3.4.4.b Virus in Naturally Infected Domestic Cats

Supplementary Data

Table S1. Histopathological lesions in the cats infected with H5N1 clade 2.3.4.4b.

	Histopathological manifestations	
**Tissues**	**Cat 1**	**Cat 2**
**Lung**		
Alveoli	Mixed inflammation and necrosis, hyaline membranes, edema, fibrin, cocci colonies	Mixed inflammation and necrosis, hyaline membranes, edema, fibrin
Bronchioles	Mixed inflammation and necrosis, epithelial hyperplasia	Mixed inflammation and necrosis
Bronchi	Mixed inflammation and necrosis	No lesion
Vasculature	Periaterial mononuclear inflammation and edema	Periaterial mononuclear inflammation and edema
Septae	Necrosis	Necrosis
**Brain**		
Location	Cerebellum & brain stem>cerebrum	Cerebrum>brain stem>cerebellum
Meninges	Mononuclear infiltrates	Mononuclear infiltrates
Parenchyma (NOS)	Random foci of necrosis with suppurative inflammation, gliosis, edema, neuronal necrosis, perivascular mononuclear infiltrates	Random foci of necrosis with suppurative inflammation, gliosis, edema, neuronal necrosis, perivascular mononuclear infiltrates
Ependyma	No lesion	No lesion
Choroid plexus	No tissue	No tissue
**Heart**		
Myocardium	Ventricular subendomyocardial myocyte necrosis and mixed inflammation	No lesion
**Kidney**		
Tubules	No lesion	No lesion
Glomeruli	No lesion	No lesion
**Liver**		
Midzonal	Random foci of necrosis with mixed inflammation	No lesion
**GI**		
Esophagus	No tissue	No tissue
Stomach	No lesion	No lesion
Small intestine	Mild, scattered, submucosal periglandular and submucosal and muscularis perivascular mononuclear infiltrates	Mild, scattered, submucosal periglandular and submucosal and muscularis perivascular mononuclear infiltrates
Large intestine	Mild, scattered, submucosal and muscularis perivascular mononuclear infiltrates	Mild, scattered, submucosal and muscularis perivascular mononuclear infiltrates
**Adrenal gland**		
Cortical	No lesion	No lesion
Medullary	No lesion	No lesion
**Pancreas**		
Endocrine	No tissue	No lesion
Exocrine	No tissue	Mononuclear infiltrates
Ductal	No tissue	Mononuclear infiltrates
**Thyroid gland**	No lesion	Mononuclear infiltrates
**Parathyroid gland**	No lesion	No tissue
**Spleen**	Marginal hyperemia	Follicular lymphoid depletion, marginal hyperemia
**Lymph nodes**	Increased cortical and medullary sinus tingible body macrophages	No tissue
**Skeletal muscle**	No lesion	No lesion

Table S2. Result of the hierarchical likelihood ratio test (hLRT) using the fixed topology given as input. Null hypothesis=the current best-fit model, Null hypothesis ln(L)=loglikelihood of the best tree using the null hypothesis model, Alternative hypothesis=the new model that is compared to the null hypothesis model, Alternative hypothesis ln(L)=loglikelihood of the best tree using the alternative hypothesis model, Δ=difference in loglikelihood between alternative hypothesis and null hypothesis, Distribution=the distribution to test Δ against, p-value=the p-value of Δ when testing against the distribution in Distribution, Outcome=outcome of the test.

**Null hypothesis**	**Null hypothesis ln(L)**	**Alternative hypothesis**	**Alternative hypothesis ln(L)**	**Δ**	**Distribution**	**p-value**
**JC**	-15715.1	F81	-15615.9	198.4022	χ-squared with 3 degree(s) of freedom	<0.0001
**F81**	-15615.9	HKY	-14848.4	1534.917	χ-squared with 1 degree(s) of freedom	<0.0001
**HKY**	-14848.4	GTR	-14783.5	129.8734	χ-squared with 4 degree(s) of freedom	<0.0001
**GTR**	-14783.5	GTR + G	-14272.5	1021.947	50:50 mix of χ-squared with 1 degree(s) of freedom and χ-squared with 0 degree(s) of freedom	<0.0001

Table S3. Bayesian information criterion test-based analyses of nucleotide substitution models. The model with the lowest BIC value is considered to be the best-fit model. The columns represent the following values: Model name=model tested, ln(L)=loglikelihood of the best tree found, K=number of parameters, BIC=the Bayesian information criterion, computed using the formula BIC=-2ln(L)+Kln(n), Δ=difference between this model's BIC value and the BIC value of the best model. Rate variation (4 categories) is indicated by +G in the model name, and +T in the model name denotes topology variation.

**Model name**	**ln(L)**	**K**	**BIC**	**Δ**
**GTR + G + T**	-14227.9	2897	50016.71	0
**HKY + G + T**	-14288.2	2893	50107.57	90.8591
**K80 + G + T**	-14368.8	2890	50246.35	229.6375
**GTR + T**	-14732.9	2896	51019.28	1002.569
**HKY + T**	-14794.2	2892	51112.17	1095.461
**K80 + T**	-14882	2889	51265.38	1248.667
**F81 + G + T**	-15056.6	2892	51636.98	1620.271
**JC + G + T**	-15142.2	2889	51785.74	1769.029
**F81 + T**	-15547.2	2891	52610.72	2594.009
**JC + T**	-15646.5	2888	52786.83	2770.116

Table S4: Minimum theoretical information criterion-based analyses of nucleotide substitution models. The model with the lowest AIC value is considered to be the best-fit model. The columns represent the following values: Model name=model tested, ln(L)=loglikelihood of the best tree found, K=number of parameters, n/K=ratio between the length of the alignment, n, and number of parameters. AIC=the Akaike information criterion, computed using the formula AIC=-2ln(L)+2K, Δ=difference between this model's AIC value and the AIC value of the best model. Rate variation (4 categories) is indicated by +G in the model name, and +T in the model name denotes topology variation.

**Model name**	**ln(L)**	**K**	**n/K**	**AIC**	**Δ**
**GTR + G + T**	-14227.9	2897	0.59	34249.81	0
**HKY + G + T**	-14288.2	2893	0.59	34362.44	112.6291
**K80 + G + T**	-14368.8	2890	0.59	34517.54	267.735
**GTR + T**	-14732.9	2896	0.59	35257.82	1008.012
**HKY + T**	-14794.2	2892	0.59	35372.48	1122.673
**K80 + T**	-14882	2889	0.59	35542.01	1292.207
**F81 + G + T**	-15056.6	2892	0.59	35897.29	1647.483
**JC + G + T**	-15142.2	2889	0.59	36062.38	1812.569
**F81 + T**	-15547.2	2891	0.59	36876.47	2626.664
**JC + T**	-15646.5	2888	0.59	37068.91	2819.098

Supplementary Figures

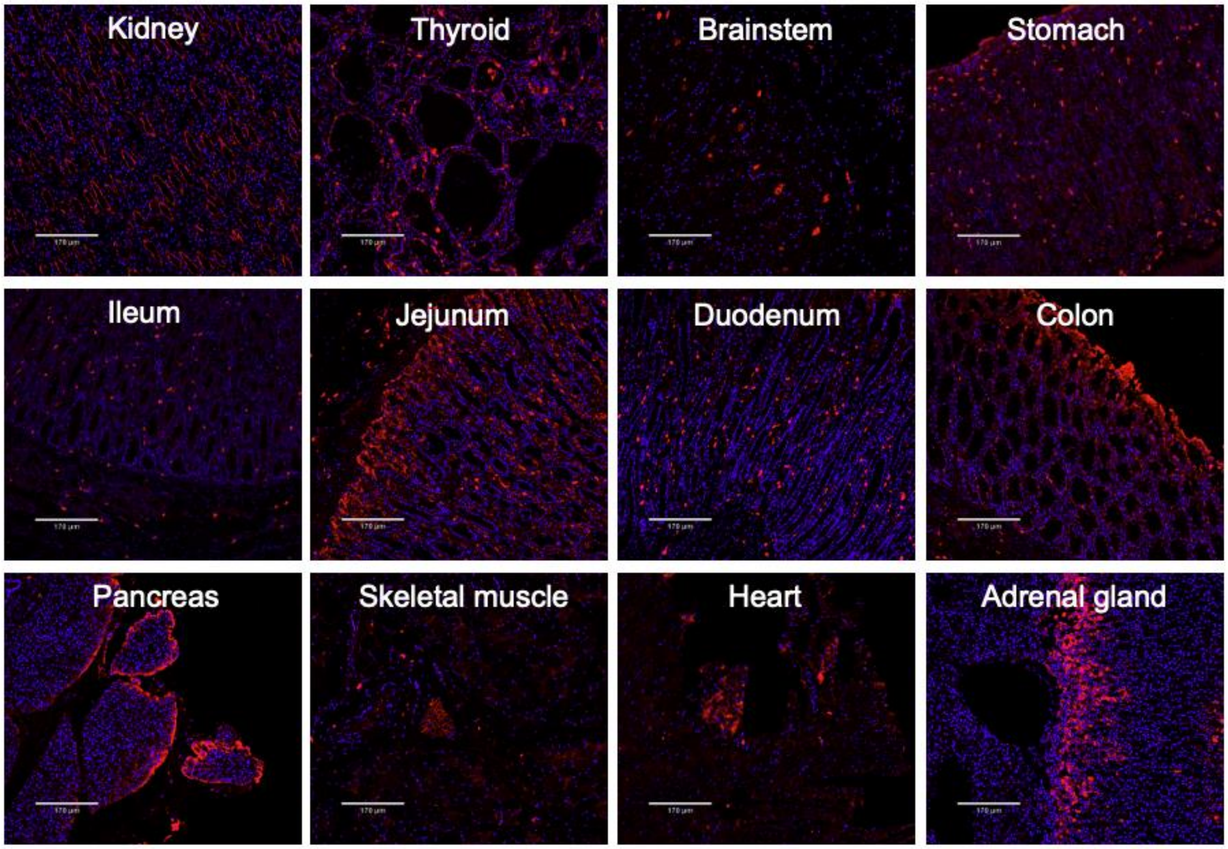

Figure S1. IAV H5N1 nucleoprotein staining in H5N1 clade 2.3.4.4b infected cat tissues. Immunohistochemistry revealed the presence of IAV H5N1 nucleoprotein in infected cat tissues – kidney, thyroid, brainstem, stomach, ileum, jejunum, duodenum, colon, pancreas, skeletal muscle, heart, and adrenal gland. Tissues were primarily stained with an anti-Influenza A virus nucleoprotein antibody, followed by a secondary goat anti-mouse IgG H&L (Alexa Fluor® 647) antibody (red) and DAPI nuclear stain (blue). Scale bar = 170 µm.

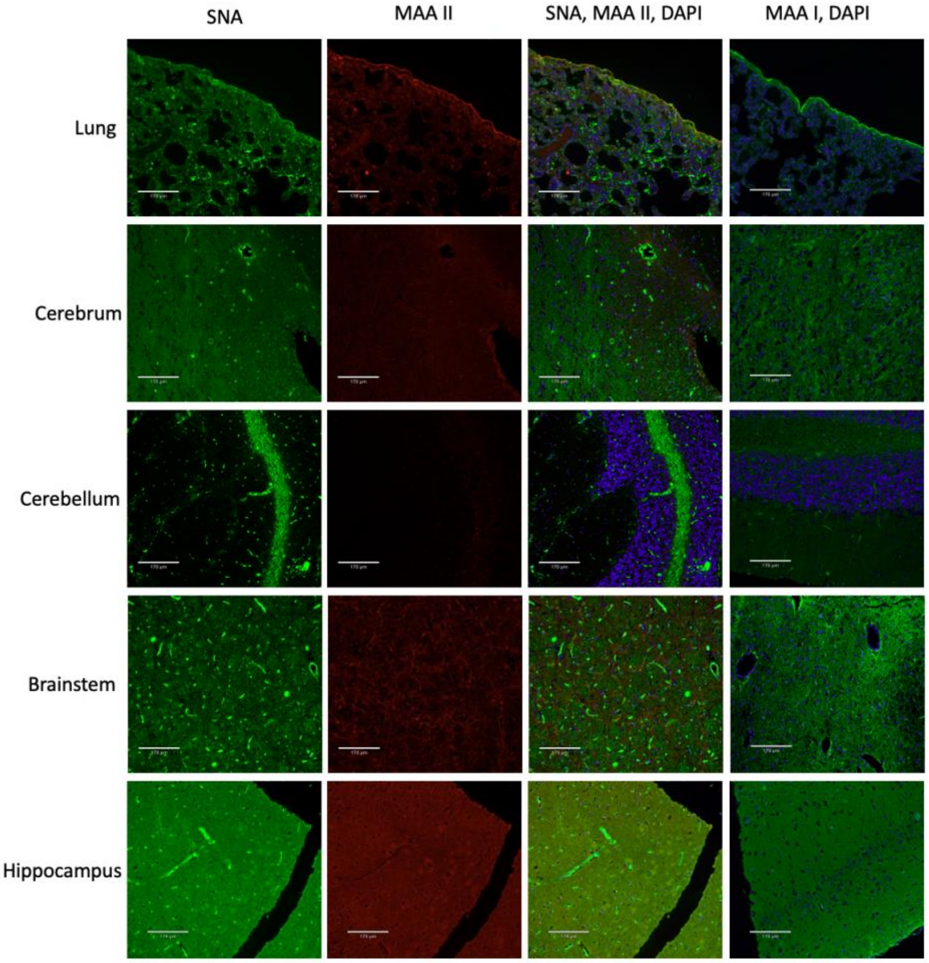

Figure S2. Expression of SA α2,3-Gal and SA α2,6-Gal receptors in IAV H5N1 clade 2.3.4.4b infected cat tissues. The composite fluorescent images reveal expression of SA α2,3-Gal (red) and SA α2,6-Gal (green) influenza receptors in the lung, cerebrum, cerebellum, and brainstem of IAV H5N1 infected cat tissue. While all the tissues showed expression of both receptors, the SA α2,6-Gal (green) receptors were expressed more than SA α2,3-Gal (red). Tissue sections were stained with FITC-labeled SNA (SA α2,6-Gal) lectin, FITC-labeled MAA I (SA α2,3-Gal), biotinylated MAA II (SA α2,3-Gal), and DAPI nuclear stain (blue). Scale bar = 170 µm**.**

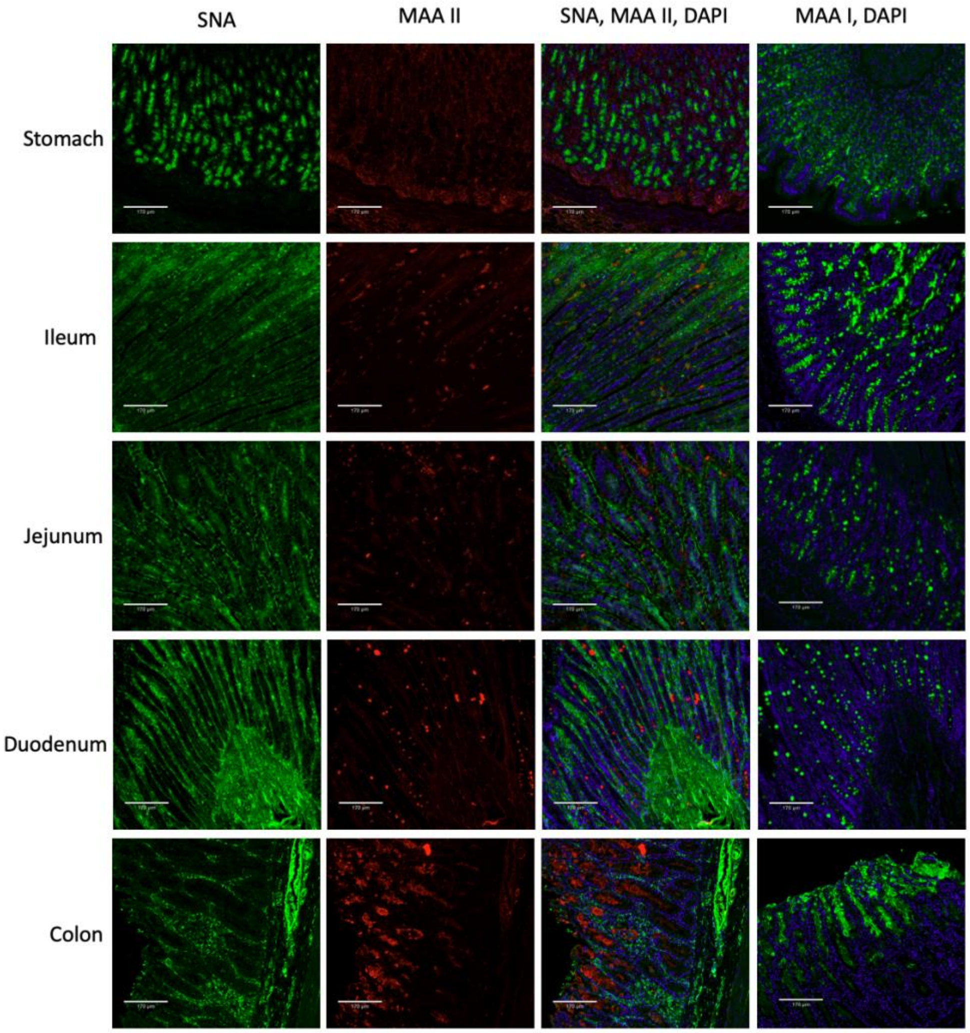

Figure S3. Expression of SA α2,3-Gal and SA α2,6-Gal receptors in IAV H5N1 clade 2.3.4.4b infected cat gastrointestinal tissues. The composite fluorescent images reveal expression of SA α2,3-Gal (red) and SA α2,6-Gal (green) influenza receptors in the stomach, ileum, jejunum, and duodenum of IAV H5N1 infected cat tissue. Tissue sections were stained with FITC-labeled SNA (SA α2,6-Gal) lectin, FITC-labeled MAA I (SA α2,3-Gal), biotinylated MAA II (SA α2,3-Gal), and DAPI nuclear stain (blue). Scale bar = 170 μm.

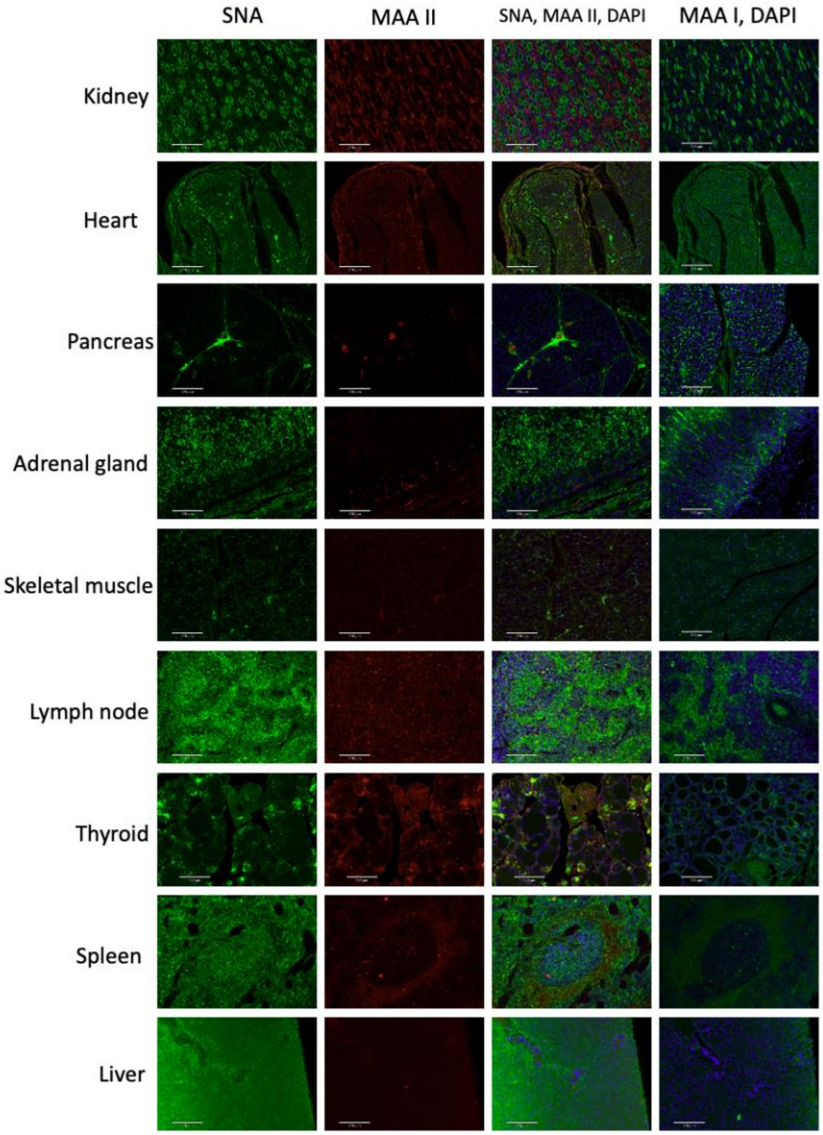

Figure S4. Expression of SA α2,3-Gal and SA α2,6-Gal receptors in various IAV H5N1 clade 2.3.4.4b infected cat tissues. The composite fluorescent images reveal the expression of SA α2,3-Gal (red) and SA α2,6-Gal (green) influenza receptors in the kidney, heart, pancreas, adrenal gland, skeletal muscle, lymph node, and thyroid. Tissue sections were stained with FITC-labeled SNA (SA α2,6-Gal) lectin, FITC-labeled MAA I (SA α2,3-Gal), biotinylated MAA II (SA α2,3-Gal), and DAPI nuclear stain (blue). Scale bar = 170 µm.

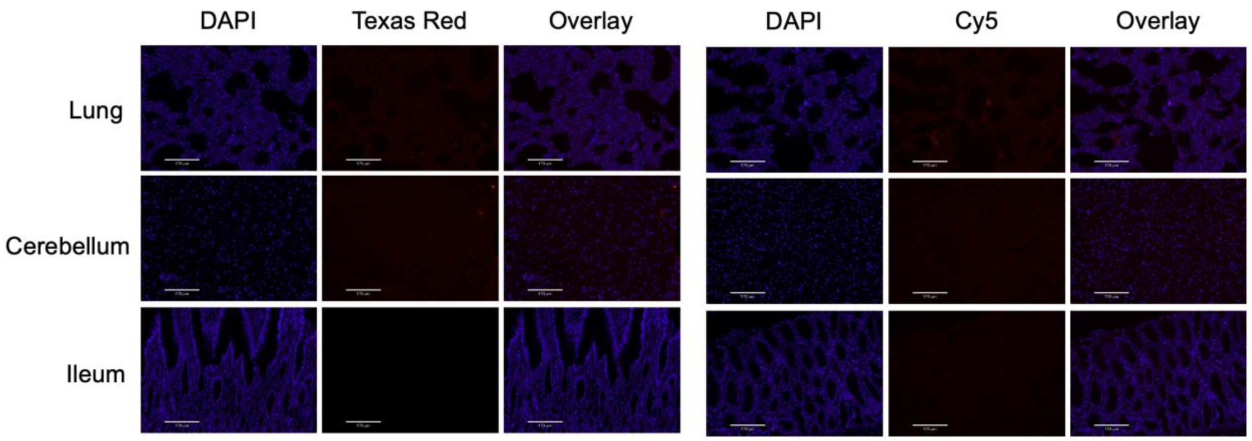

Figure S5. Negative control cat tissues exhibit minimal background noise. The negative control for lectin histochemistry, which included omission of the lectin and incubation with Streptavidin, Alexa Fluor™ 594 Conjugate, analyzed in Texas Red channel, demonstrated minimal background across various tissues such as the lung, cerebellum, and ileum (A, B, C). For the nucleoprotein histochemistry and virus binding assay controls, where no primary antibody and no virus/no primary antibody were used, the secondary antibody, Goat Anti-Mouse IgG H&L (Alexa Fluor® 647), analyzed in the Cy5 channel, also showed minimal background noise (D, E, F). Images were captured using an Echo fluorescent microscope at 10x magnification. Scale bar = 170 µm
